# Supplementary material for: Development of the Entorhinal Cortex Occurs via Parallel Lamination During Neurogenesis
Source: Front Neuroanat. 2021 May 5;15:663667. doi: 10.3389/fnana.2021.663667 (PMC8139189; doi:10.3389/fnana.2021.663667)
Supplement: Supplementary Figure 1 — Borders of the developing porcine entorhinal cortex (EC). Cresyl violet stained 4 or 5 coronal sections of the piriform lobe from Embryonic day (E)60 to postnatal day (P)75 depicted in a rostral to caudal series. The first section is rostral to the EC, the second section depicts the LEC occupying the EC entity, the third section includes both MEC and LEC present, the fourth section depicts the MEC occupying the entire mediolateral entity and the fifth section is the most caudal part of the piriform lobe. Dentate gyrus (DG); hippocampal area (HA); amygdala (Amyg); posterior rhinal sulcus (RHP); medial entorhinal cortex (MEC); lateral entorhinal cortex (LEC); pre-subiculum (PreS); para-subiculum (PaS), subiculum (Sub); perirhinal cortex (PER). Scale bar 1 cm. [file Data_Sheet_1.PDF]

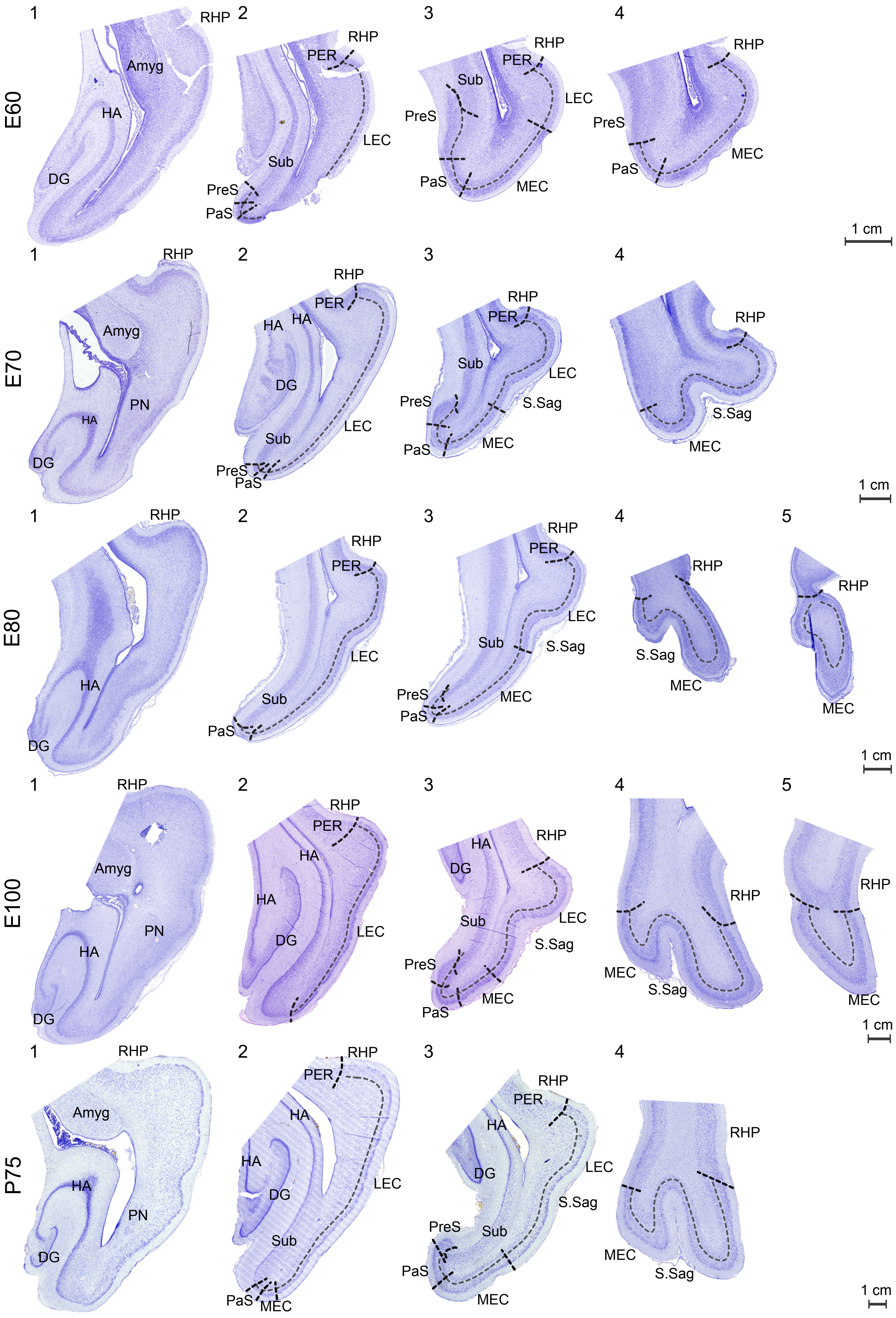

E60
1
RHP
Amyg
HA
DG
2
RHP
PER
LEC
Sub
PreS
PaS
3
RHP
PER
Sub
LEC
PreS
PaS
MEC
4
RHP
PreS
MEC
PaS
1 cm
E70
1
RHP
Amyg
PN
HA
DG
2
RHP
PER
HA
HA
DG
LEC
Sub
PreS
PaS
3
RHP
PER
Sub
LEC
S.Sag
PreS
PaS
MEC
4
RHP
S.Sag
MEC
1 cm
E80
1
RHP
HA
DG
2
RHP
PER
LEC
Sub
PaS
3
RHP
PER
LEC
Sub
S.Sag
PreS
PaS
MEC
4
RHP
S.Sag
MEC
5
RHP
MEC
1 cm
E100
1
RHP
Amyg
PN
HA
DG
2
RHP
PER
HA
HA
LEC
DG
3
HA
RHP
DG
Sub
LEC
S.Sag
PreS
PaS
MEC
4
RHP
MEC
S.Sag
5
RHP
MEC
1 cm
P75
1
RHP
Amyg
HA
PN
DG
2
RHP
PER
HA
HA
DG
LEC
Sub
PaS
MEC
3
RHP
PER
HA
DG
LEC
S.Sag
PreS
Sub
PaS
MEC
4
RHP
MEC
S.Sag
1 cm

Figure S1. Borders of the developing porcine entorhinal cortex (EC). Cresyl violet stained 4 or 5 coronal sections of the piriform lobe from Embryonic day (E)60 to postnatal day (P)75 depicted in a rostral to caudal series. The first section is rostral to the EC, the second section depicts the LEC occupying the EC entity, the third section includes both MEC and LEC present, the fourth section depicts the MEC occupying the entire mediolateral entity and the fifth section is the most caudal part of the piriform lobe. Dentate gyrus (DG); hippocampal area (HA); amygdala (Amyg); posterior rhinal sulcus (RHP); medial entorhinal cortex (MEC); lateral entorhinal cortex (LEC); pre-subiculum (PreS); para-subiculum (PaS), subiculum (Sub); perirhinal cortex (PER). Scale bar 1 cm.

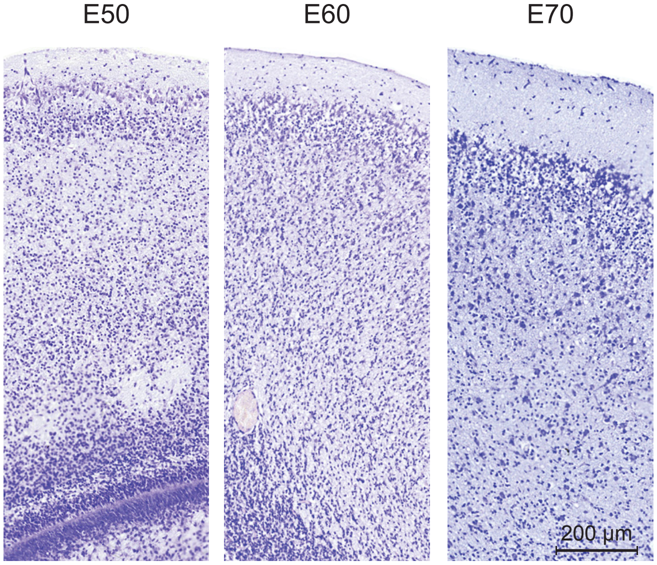

Figure S2. The morphology of the entorhinal cortex at Embryonic day (E)50 to E70. Cresyl violet staining of the developing cortex show a prominent layer or entorhinal neurons with large nuclei in the superficial layer from E50 onwards whereas, the glia cells are difficult to identify at E50 from the nissl staining alone. Scale bar 200 μm.

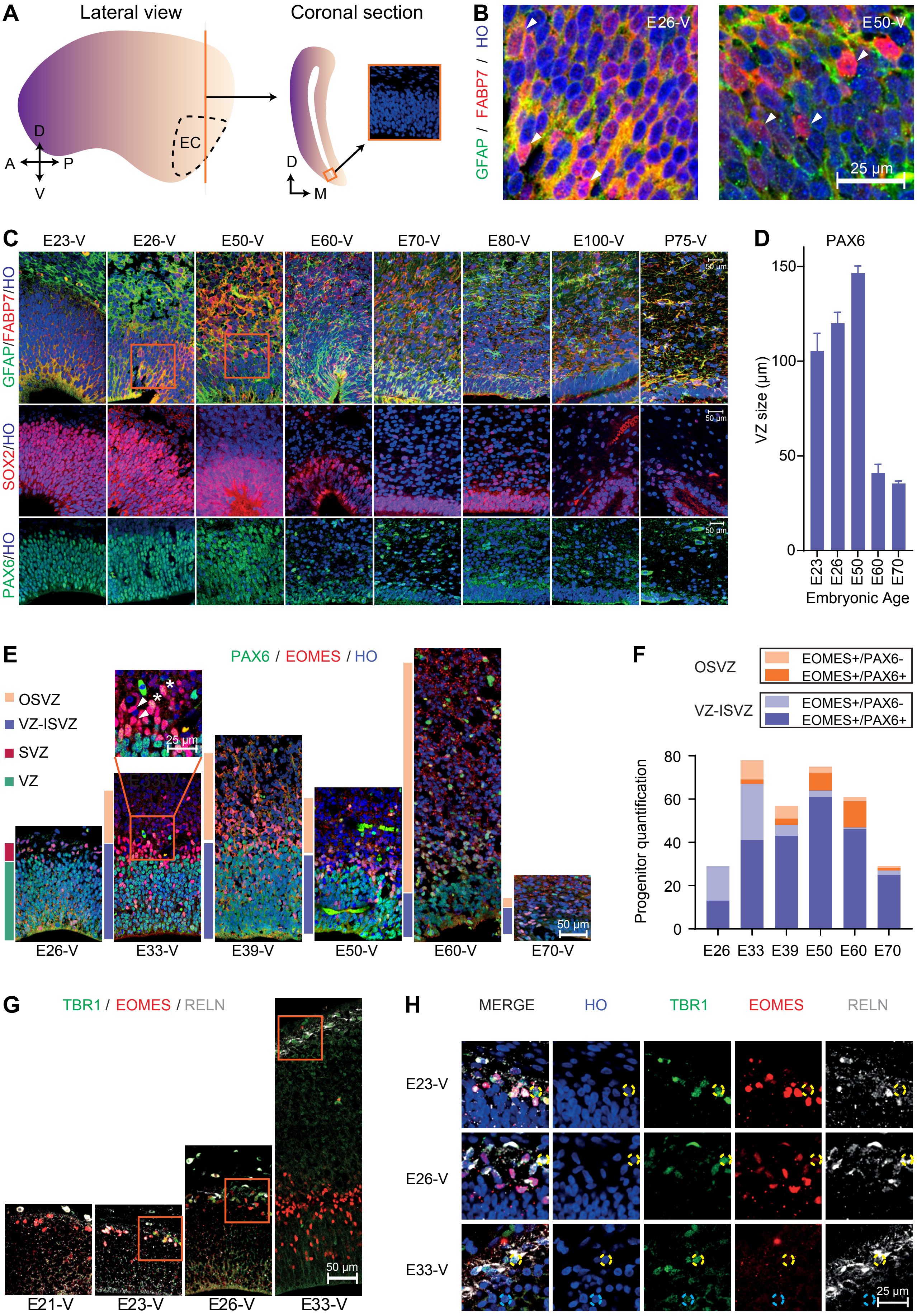

A
Lateral view
Coronal section
EC
D
A
P
V
D
M
B
GFAP / FABP7 / HO
E26-V
E50-V
25 µm
C
E23-V
E26-V
E50-V
E60-V
E70-V
E80-V
E100-V
P75-V
GFAP/FABP7/HO
SOX2/HO
PAX6/HO
50 µm
D
PAX6
VZ size (µm)
150
100
50
0
E23
E26
E50
E60
E70
Embryonic Age
E
PAX6 / EOMES / HO
OSVZ
VZ-ISVZ
SVZ
VZ
25 µm
50 µm
E26-V
E33-V
E39-V
E50-V
E60-V
E70-V
F
OSVZ
EOMES+/PAX6-
EOMES+/PAX6+
VZ-ISVZ
EOMES+/PAX6-
EOMES+/PAX6+
Progenitor quantification
80
60
40
20
0
E26
E33
E39
E50
E60
E70
G
TBR1 / EOMES / RELN
50 µm
E21-V
E23-V
E26-V
E33-V
H
MERGE
HO
TBR1
EOMES
RELN
E23-V
E26-V
E33-V
25 µm

Figure S3 Characterization of the germinal layers in the developing porcine entorhinal cortex (EC). (A) A schematic overview of the location of the characterized medial EC (MEC). Axes: D, dorsal; V, Ventral; A, Anterior; P, posterior, M, medial. Orange line depicts the anatomical location of the cut sections. **(B)** Expression of GFAP and FABP7 in the MEC ventricular zone (VZ). Scale bar 25 μm. Images are enlarged from boxes shown in C. White arrows mark co-localized expression of GFAP and FABP7. **(C)** Temporal expression of radial glia (GFAP, FABP7, PAX6, SOX2) during MEC development. Scale bar 50 μm. **(D)** Quantification of the thickness in μm of the VZ during development. Error bars represent SD. **(E)** Expression of EOMES and PAX6 in the EC. White arrow heads depict EOMES+/PAX6- cells and asterixis depict EOMES+/PAX6+ cells. Scale bar 25 μm (up) and 50μm (bottom). **(F)** Quantification of the EOMES+/PAX6+ and EOMES+/PAX6- cell populations in the germinal zone. **(G)** TBR1/EOMES/RELN expression during development. Scale bar 50 μm. **(H)** Expression of TBR1/EOMES/RELN in the marginal zone and the cortical plate, enlarged from boxes in G. Yellow circled cells are TBR1+/EOMES-/RELN- Scale bar 25 μm. (HO = Hoeschst, V = ventral telencephalon).

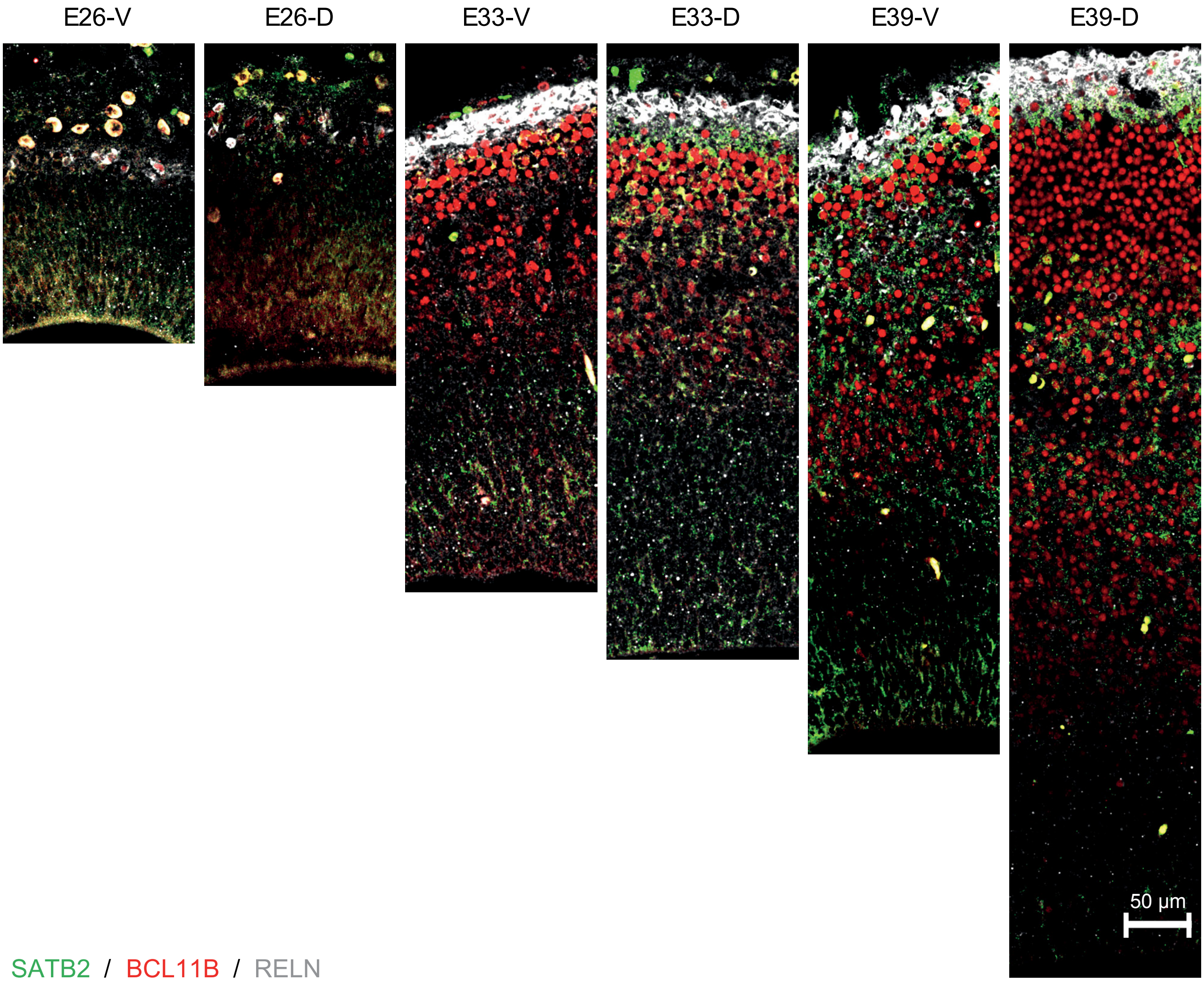

Figure S4. Comparative expression of superficial and deep layer markers in the early developing entorhinal cortex (EC) versus the dorsal, cingulate gyrus. Expression of the canonical deep layer marker (BCL11B), superficial layer marker (SATB2) and stellate cell / Cajal–Retzius cells (CR cells) marker RELN from Embryonic day (E)26 to E39 shows the prevalence of BCL11B and SATB2 from E33 onwards in the superficial marginalzone/-cortical plate and the absence of RELN at these time points within the developing cortical plate. Scale bar 50 µm.

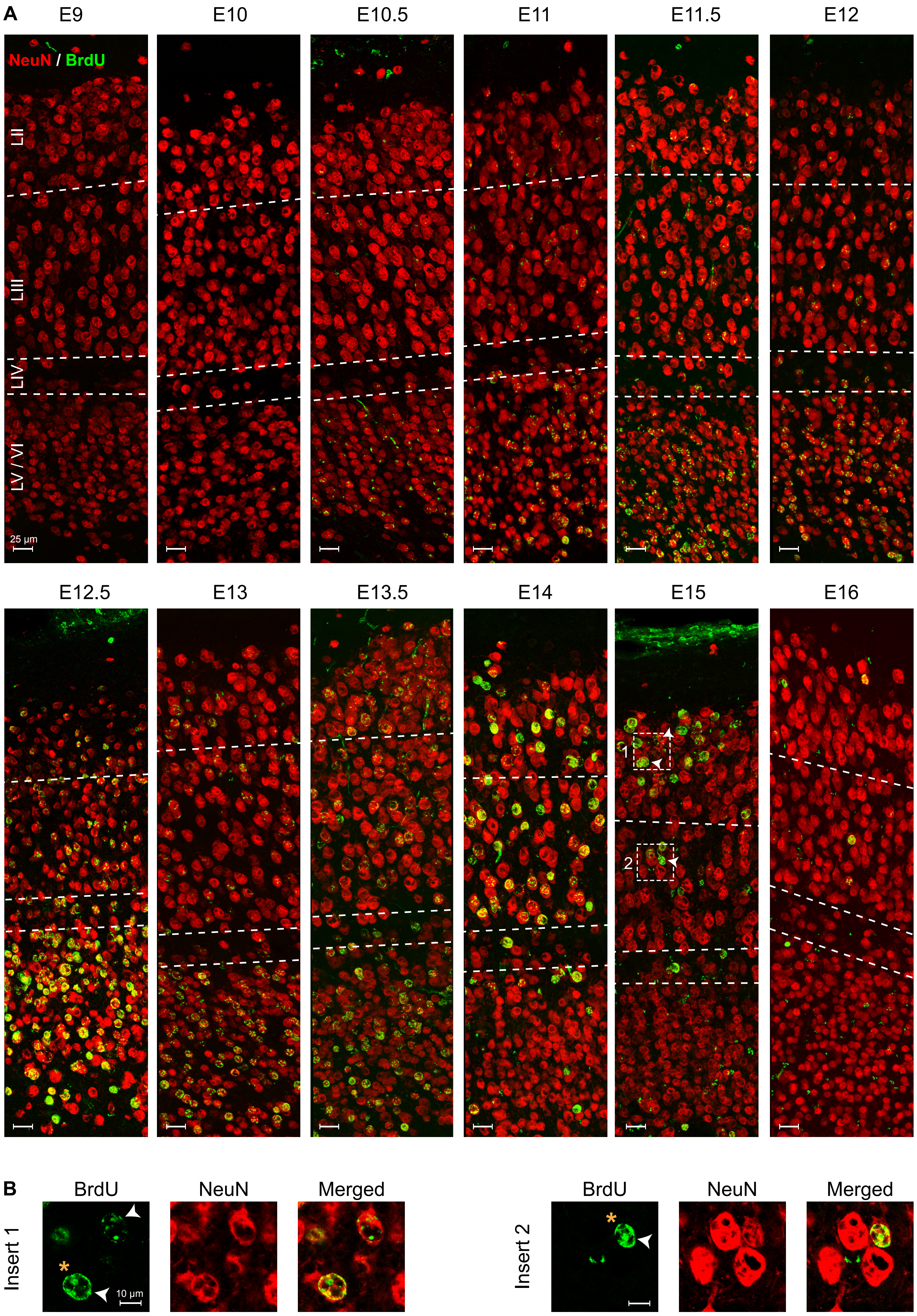
A
E9
E10
E10.5
E11
E11.5
E12
NeuN / BrdU
LII
LIII
LIV
LV / VI
25 µm
E12.5
E13
E13.5
E14
E15
E16
1
2
B
BrdU
NeuN
Merged
Insert 1
10 µm
Insert 2

Figure S5. Laminar birth dating of the medial entorhinal cortex (MEC) in the mouse. Bromodeoxyuri-dine (BrdU) labelling in combination with analysis of NeuN expression of postnatal day (P)7 MEC shows the emergence of newborn neurons from embryonic day (E)10 to E16. **(A)** Representative z-stack images from BrdU injections from E9-E16. Dotted lines represent the LII/LIII boundary and the LIV lamina dissecans. Insert boxes are shown at higher magnification in B. White arrowheads depict cells highlighted in B. Scale bar 25 μm. **(B)** High magnification of representative BrdU labelled cells with overlapping expression of NeuN in a single z-plane from LII and LIII areas in A. White arrows highlight BrdU labeled cells. Yellow asterix (*) denotes cells with strong BrdU labelling which were considered to be born at the time of BrdU injection. Scale bar 10 μm.
