# Supplementary material for: Development of the Entorhinal Cortex Occurs via Parallel Lamination During Neurogenesis
Source: Front Neuroanat. 2021 May 5;15:663667. doi: 10.3389/fnana.2021.663667 (PMC8139189; doi:10.3389/fnana.2021.663667)
Supplement: Supplementary Table 1 — Overview of porcine brains and brain sections used in the study. [file Table_1.docx]

**Supplementary Table 1. Overview of porcine brains and brain sections used in the study**

| **Brain age** | **E21** | **E23** | **E26** | **E33** | **E39** | **E50** | **E60** | **E70** | **E80** | **E100** | **P75** |
| --- | --- | --- | --- | --- | --- | --- | --- | --- | --- | --- | --- |
| **No. of brains IHC** | 3 | 3 | 3 | 3 | 3 | 3 | 3 | 3 | 3 | 3 | 3 |
| **Coronal serially sectioned brains with Nissl staining** | - | - | - | - | - | 1 | 1 | 1 | 1 | 1 | 1 |
| **No. of brains DTI MRI** | - | - | - | - | - | - | 3 | 3 | 3 | 3 | 3 |
| **No. of sections per brain in IHC** | 1 | 1 | 1 | 1 | 1 | 1 | 1 | 1 | 1 | 1 | 1 |
| **No. of sections per brain in OLIG2 and EOMES/PAX6 quantification** |  |  |  |  |  | 3 | 3 | 3 | 3 | 3 | 3 |

Abbreviations: E, embryonic day; IHC, immunohistochemistry; No. Number; P: postnatal day

**Supplementary Table 2: Overview of mouse brains, litters and sections used in the study**

| **BrdU injection at** | **E8** | **E8.5** | **E9** | **E9.5** | **E10** | **E10.5** | **E11** | **E11.5** | **E12** | **E12.5** | **E13** | **E13.5** | **E14** | **E15** | **E16** |
| --- | --- | --- | --- | --- | --- | --- | --- | --- | --- | --- | --- | --- | --- | --- | --- |
| **Brains collected at** | P7 | P7 | P7 | P7 | P7 | P7 | P7 | P7 | P7 | P7 | P7 | P7 | P7 | P7 | P7 |
| **No. of brains** | 3 | 3 | 3 | 3 | 3 | 3 | 3 | 3 | 3 | 3 | 3 | 3 | 3 | 3 | 3 |
| **No. of litters** | 1 | 1 | 1 | 1 | 1 | 1 | 1 | 1 | 1 | 1 | 1 | 1 | 1 | 1 | 1 |
| **No. of sections per brain** | 1 | 1 | 1 | 1 | 1 | 1 | 1 | 1 | 1 | 1 | 1 | 1 | 1 | 1 | 1 |

**Supplementary Table 3. Overview of primary antibodies and dilutions used**

| **Primary antibodies** | | | | |
| --- | --- | --- | --- | --- |
| **Species of tissue used** | **Epitope** | **Species** | **Dilution** | **Source and Catalogue Number** |
| Mouse | BrdU | Mouse | 1:500 | BD Biosciences, #347580 |
| Mouse | NEUN | Rabbit | 1:1000 | Abcam, #Ab177487 |
| Mouse/Pig | SATB2 | Mouse | 1:750 | Abcam, #Ab51502 |
| Mouse/Pig | BCL11B | Rat | 1:750 | Abcam, #Ab18465 |
| Mouse/Pig | RELN | Goat | 1:500 | RD Systems, #AF3820 |
| Pig | OLIG2 | Rabbit | 1:500 | Merck Millipore, #AB9610 |
| Pig | TBR1 | Rabbit | 1:200 | Abcam, #AB31940 |
| Pig | EOMES | Sheep | 1:40 | R and D System,s #AF6166 |
| Pig | GFAP | Mouse | 1:500 | DAKO, # Z0334 |
| Pig | FABP7 | Rabbit | 1:50 | Santa Cruz, #sc-374588 |
| Pig | SOX2 | Mouse | 1:100 | R and D Systems, #MAB2018 |
| Pig | PAX6 | Rabbit | 1:200 | Biolegend, # 901301 |
| Pig | Parvalbumin | Mouse | 1:500 | Sigma-Aldrich, #P3088 |
| Pig | Calbindin | Rabbit | 1:500 | Millipore, #AB1778 |

**Supplementary Table 4. Overview of secondary and negative control antibodies and dilutions used**

| **Secondary antibodies** | | | | |
| --- | --- | --- | --- | --- |
| **Alexa Fluor/Control** | **Epitope** | **Species** | **Dilution** | **Source and Catalogue number** |
| 488 | Mouse IgG | Donkey | 1:500 | Abcam, #Ab150105 |
| 488 | Rat IgG | Donkey | 1:500 | Invitrogen, #A21208 |
| 546 | Mouse IgG | Donkey | 1:500 | Invitrogen #A10036 |
| 647 | Rabbit IgG | Donkey | 1:500 | Abcam, #Ab150075 |
| 647 | Goat IgG | Donkey | 1:500 | Invitrogen, #A21448 |
| 647 | Sheep IgG | Donkey | 1:500 | Invitrogen, #A21448 |
| Negative control | IgG | Rabbit | 1:40 | DAKO, #X0936 |
| Negative control | IgG1 | Mouse | 1:40 | DAKO, #X0931 |
| Negative control | IgG2a | Mouse | 1:200 | DAKO, #X0943 |

**Supplementary Table 5: Raw data for quantification of neurons in mouse brains**

| **Layer** | **Cell counted** | **Analyzed brains** | | | | | | | | | | | | | | | | | | | | | | | | | | | | | |
| --- | --- | --- | --- | --- | --- | --- | --- | --- | --- | --- | --- | --- | --- | --- | --- | --- | --- | --- | --- | --- | --- | --- | --- | --- | --- | --- | --- | --- | --- | --- | --- |
|  |  | **E10.5A** | **E10.5B** | **E10.5C** | **E11A** | **E11B** | **E11C** | **E11.5A** | **E11.5b** | **E11.5C** | **E12A** | **E12B** | **E12C** | **E12.5A** | **E12.5B** | **E12.5C** | **E13A** | **E13B** | **E13C** | **E13.5A** | **E13.5B** | **E13.5C** | **E14A** | **E14B** | **E14C** | **E15A** | **E15B** | **E15C** | **E16A** | **E16B** | **E16C** |
| **L2** | **BrdU** | 0 | 0 | 1 | 2 | 3 | 2 | 3 | 2 | 1 | 3 | 2 | 2 | 9 | 8 | 5 | 10 | 13 | 7 | 15 | 6 | 8 | 9 | 11 | 11 | 22 | 23 | 24 | 3 | 4 | 3 |
|  | **NeuN** | 50 | 50 | 51 | 80 | 57 | 44 | 87 | 78 | 35 | 66 | 66 | 48 | 79 | 56 | 53 | 74 | 103 | 53 | 101 | 46 | 54 | 49 | 46 | 38 | 90 | 99 | 104 | 79 | 68 | 63 |
| **L3** | **BrdU** | 0 | 0 | 0 | 2 | 3 | 3 | 2 | 2 | 1 | 2 | 6 | 3 | 11 | 15 | 7 | 8 | 7 | 8 | 15 | 8 | 10 | 27 | 30 | 11 | 7 | 7 | 8 | 3 | 0 | 0 |
|  | **NeuN** | 100 | 100 | 100 | 128 | 104 | 118 | 127 | 109 | 77 | 141 | 108 | 81 | 105 | 118 | 92 | 90 | 60 | 83 | 121 | 90 | 87 | 86 | 84 | 66 | 105 | 78 | 94 | 117 | 84 | 1 |
| **L5/6** | **BrdU** | 5 | 8 | 5 | 28 | 35 | 34 | 38 | 34 | 28 | 31 | 34 | 25 | 79 | 80 | 61 | 73 | 42 | 46 | 63 | 45 | 40 | 8 | 4 | 1 | 3 | 4 | 3 | 1 | 1 | 0 |
|  | **NeuN** | 129 | 167 | 155 | 188 | 196 | 199 | 261 | 223 | 183 | 220 | 179 | 143 | 195 | 183 | 161 | 198 | 137 | 139 | 217 | 143 | 128 | 205 | 123 | 149 | 178 | 210 | 223 | 232 | 228 | 1 |

**Supplementary Table 6.** Connectivity of neuronal tracts to/from the lateral entorhinal cortex (LEC) and the medial entorhinal cortex (MEC) in the pig at postnatal day (P)75. The average connectivity from three analyzed brains is shown with the standard deviation in brackets. Abbreviations: AM, amygdala; CN, caudate nucleus, DG, dentate gyrus; HC, dorsal hippocampal commissure; LEC, lateral entorhinal cortex; LOT, lateral olfactory tract; MEC, medial entorhinal cortex; Nucleus accumbens, NAc; PUT, putamen; SUB, subiculum.

|  | **Total Tracts** | **MEC** | **LEC** | **AM** | **CA1** | **CA3** | **DG** | **PUT** | **SUB** | **LOT** | **CN** | **NAc** | **HC** | **Other** |
| --- | --- | --- | --- | --- | --- | --- | --- | --- | --- | --- | --- | --- | --- | --- |
| **MEC** | 6205 |  | 106  (1.71) | 730.3  (11.77) | 91.3  (1.47) | 49.3  (0.79) | 47  (0.76) | 4.7  (0.08) | 534  (8.61) | 3.3  (0.05) | 9.0  (0.15) | 1.0  (0.02) | 11.7  (0.19) | 4617.3  (74.41) |
| **LEC** | 4516.3 | 106  (2.35) |  | 468.7  (10.38) | 5.3  (0.12) | 10  (0.22) | 2.7  (0.06) | 59  (1.31) | 1  (0.02) | 14.3  (0.32) | 75.3  (1.67) | 0  (0) | 0  (0) | 3774  (83.56) |
